# Keratinocyte serum-free medium maintains long-term liver gene expression and function in cultured rat hepatocytes by preventing the loss of liver-enriched transcription factors

**DOI:** 10.1016/j.biocel.2006.10.017

**Published:** 2007

**Authors:** Wan-Chun Li, Kate L. Ralphs, Jonathan M.W. Slack, David Tosh

**Affiliations:** Centre for Regenerative Medicine, Department of Biology and Biochemistry, University of Bath, Claverton Down, Bath BA2 7AY, UK

**Keywords:** W, Williams’ medium E, KSFM, keratinocyte serum-free medium, Dex, dexamethasone, EGF or E, human epidermal growth factor, PGE or P, pituitary gland extract, C/EBP, CCAAT/enhancer-binding protein, HNF, hepatocyte nuclear factor, RXR, retinoid X receptor, PXR, pregnane X receptor, CYPs, cytochrome P450 proteins, GS, glutamine synthetase, CPS, carbamoylphosphate synthetase, Cx, connexin, RT-PCR, reverse transcription polymerase chain reaction, UGT, UDP-glucuronosyltransferase, CAR, constitutive active receptor, DMSO, dimethyl sulfoxide, Hepatocyte culture, Keratinocyte serum-free media, Liver-enriched transcription factors

## Abstract

Freshly isolated hepatocytes rapidly lose their differentiated properties when placed in culture. Therefore, production of a simple culture system for maintaining the phenotype of hepatocytes in culture would greatly facilitate their study. Our aim was to identify conditions that could maintain the differentiated properties of hepatocytes for up to 28 days of culture. Adult rat hepatocytes were isolated and attached in Williams’ medium E containing 10% serum. The medium was changed to either fresh Williams’ medium E or keratinocyte serum-free medium supplemented with dexamethasone, epidermal growth factor and pituitary gland extract. The hepatic phenotype was then analysed using RT-PCR, immunohistochemistry, Western blotting and assays of liver function. Cells cultured in keratinocyte serum-free medium supplemented with dexamethasone, epidermal growth factor and pituitary gland extract maintained their phenotype for 3–4 weeks, based on expression of liver proteins, ureagensis and response to xenobiotics. In contrast, hepatocytes cultured in Williams’ medium E rapidly lost the expression of liver proteins after 3 days. Cells cultured in keratinocyte serum-free medium supplemented with dexamethasone, epidermal growth factor and pituitary gland extract maintained their expression of liver-enriched transcription factors (C/EBPα and β, HNF4α and RXRα) while expression was either lost or reduced in cells cultured in Williams’ medium E. These results suggest that keratinocyte serum-free medium supplemented with dexamethasone, epidermal growth factor and pituitary gland extract can maintain the hepatic phenotype for a prolonged period and that this is probably related to the continued expression of the liver-enriched transcription factors.

## 1 Introduction

The primary culture of hepatocytes from rodents constitutes an attractive model system for the study of liver function. However, a major limitation to such a model is the rapid and irreversible loss of differentiated hepatic functions in culture. De-differentiation is reflected not only in decreased liver-specific functions, but there is also an alteration of morphology: the cells flatten, depolarize, and lose many of the surface characteristics of normal hepatocytes *in vivo*. The mechanisms responsible for the loss of differentiated properties probably involve the downregulation of transcription factors involved in liver-specific gene expression (Padgham et al., 1993). The loss of differentiated functions has been attributed to the change in environmental conditions (extracellular matrix, hormonal conditions) following cell isolation (Padgham & Paine, 1993). The loss of the differentiated phenotype is most apparent in the rapid decline of total cytochrome P450s (CYPs) after isolation and in culture, particularly the CYP3A1 isoform (Padgham et al., 1993). Studies of liver function are therefore generally confined to the first few days of culture and this precludes longer-term studies. Conventional approaches to maintaining the differentiated properties of isolated hepatocytes in culture include supplementation of the medium with hormones such as dexamethasone (Dex) (Agius, Chowdhury, & Alberti, 1986; Enat et al., 1984), co-factors such as nicotinamide, pyruvate, DMSO and phenobarbital (LeCluyse, Bullock, Parkinson, & Hochman, 1996; Waxman, Morrissey, Naik, & Jauregui, 1990); the application of extracellular matrix components (Bissell, Arenson, Maher, & Roll, 1987; Gomez-Lechon et al., 1998) and co-culture with non-parenchymal epithelial cell-types (Gomez-Lechon et al., 1998, Rogiers and Vercruysse, 1993; Vallette et al., 1998).

In the present study we have introduced a new medium for the long-term culture of differentiated hepatocytes. This is keratinocyte serum-free medium (KSFM). It was tested both alone, and in combination with supplements (dexamethasone, EGF and pituitary gland extract), in comparison with the standard Williams’ medium E, and was used to maintain rat hepatocytes in culture for up to 28 days. We performed immunohistochemical, Western blotting and RT-PCR analysis of the cells under the different culture conditions. We chose hepatic markers which are known to be either rapidly switched off after hepatocyte isolation or are important for detoxification. Expression of a particular gene or protein is normally taken as an indication of intact liver function. However, it is difficult to know simply from expression of a gene or protein that the associated function remains intact. For this reason we also assayed for hepatocyte ureagenesis, glycogen synthesis and response to xenobiotics. Lastly, to gain some mechanistic insight we determined the expression of several liver-enriched transcription factors. Our results suggest that KSFM, in combination with dexamethasone, EGF and pituitary gland extract can maintain the liver phenotype for between 21 and 28 days and it is likely that the ability to do this depends on the sustained expression of liver-enriched transcription factors.

## 2 Materials and methods

### 2.1 Materials

Collagenase was obtained from Worthington Biochemical Corporation, dexamethasone, penicillin/streptomycin antibiotics, l-glutamine, Williams’ medium E and phenobarbital were purchased from Sigma Chemical Co. (St. Louis, MO). Fungizone (amphotericin B) and Keratinocyte serum-free medium (KSFM) and supplements, recombinant human epidermal growth factor and bovine pituitary extract at final concentrations of 5 ng/ml and 50 μg/ml, respectively, were obtained from GIBCO™. Sources of other chemicals and media were as described previously (Shen, Slack, & Tosh, 2000; Tosh, Shen, & Slack, 2002).

### 2.2 Rat hepatocyte isolation and culture

Male Wistar rats (270–330 g) were obtained from the Animal House of the University of Bath. Hepatocytes were isolated by the two-step collagenase perfusion technique as described previously (Tosh, Alberti, & Agius, 1988). Calcium-free EDTA perfusion medium was freshly prepared with the following composition: 0.05% (w/v) KCl (Fisher Scientific) in 10 mM 4-(2-hydroxyethyl)-1-piperazineethanesulfonic acid (HEPES, Sigma) buffer, 5 mM d-glucose (Sigma), 200 μM EDTA (Sigma) and 1/1000 v/v phenol red (Sigma) all in sterile phosphate buffered saline (PBS, pH7.4) The liver was perfused via the portal vein for approximately 10 min at a flow rate of 30–35 ml/min and then replaced with a collagenase-containing perfusion medium [composition: 20 mM HEPES buffer, 5 mM d-glucose, 1 mM CaCl_2_ (Fisher Scientific), 0.001% (v/v) phenol red (Sigma) all in sterile PBS (pH 7.4) and contained collagenase II (0.33 mg/ml, w/v)]. Approximately 150 ml was delivered to the liver for 10–15 min (with recirculation) at a flow rate of 30–35 ml/min. Following collagenase perfusion, the liver was removed and hepatocytes dissociated using fine forceps. The cells were filtered through a 70 μm filter. Cells were washed three times (50 × *g* for 2 min) in medium [20 mM HEPES buffer, 5 mM d-glucose, 1 mM CaCl_2_ (Fisher scientific), 0.001% (v/v) phenol red (Sigma) all in sterile saline at pH 7.4]. Using trypan blue (Sigma), the cell viability was determined to be approximately 85%. Approximately 3–5 × 10^5^ rat hepatocytes were initially placed in 35 mm culture dishes in hepatocyte attachment medium (Williams’ medium E containing 10 U/ml penicillin, 100 μg/ml streptomycin,  2mM l-glutamine, and 10% foetal bovine serum). After 6–8 h, the attachment medium was removed and replaced with either serum-free Williams’ medium E or KSFM medium. Both media contained 10 U/ml penicillin, 100 μg/ml streptomycin, 2 mM l-glutamine, 50 μg/ml gentamicin, 100 ng/ml Fungizone with or without the indicated supplements. The composition with supplements was as follows: Williams’ medium E only (W), Williams’ medium E plus 1 μM Dex (WD), Williams’ medium E plus 5 ng/ml EGF (WE), Williams’ medium E plus 1 μM Dex plus 50 μg/ml pituitary gland extract (WDP), Williams’ medium E plus 1 μM Dex plus 5 ng/ml EGF (WDE), Williams’ medium E plus supplements (both 5 ng/ml EGF and 50 μg/ml pituitary gland extract) (WS) and Williams’ medium E plus supplements (5 ng/ml EGF and 50 μg/ml pituitary gland extract) plus 1 μM Dex (WDS); KSFM (K), KSFM plus 1 μM Dex (KD), KSFM plus 50 μg/ml pituitary gland extract (KP), KSFM plus 5 ng/ml EGF (KE), KSFM plus supplement (KS) and KSFM plus supplement plus 1 μM Dex (KDS).

### 2.3 Immunostaining and antisera

For immunofluorescence staining, cells cultured on glass coverslips were rinsed with PBS to remove any excess medium and then fixed with 4% PFA for 20–30 min. The cells were washed twice in PBS and kept at 4 °C prior to staining. Cells were immunostained as described previously (Shen et al., 2000). The coverslips were mounted with Gel/Mount aqueous mounting medium (Biomeda, Foster City, CA). The primary and secondary antibodies used for immunofluorescence staining are listed in Table 1. For the detection of connexin 32 protein, the cells were fixed in acetone/methanol (1:1, v/v) then incubated in 1× citrate buffer (Lab Vision Corporation, CA) at 37 °C for 30 min before blocking and staining.

**Table 1:** 

	Species	Dilution factor	Supplier
Primary antibody			
Albumin	Rabbit	1/300	Sigma
α1-Antitrypsin	Rabbit	1/100	Sigma
Apolipoprotein B (ApoB)	Mouse	1/100	Chemicon International
Carbamoyl-phosphate synthetase I (CPS1)	Rabbit	1/300	A gift from Dr. Wouler H. Lames, University of Amsterdam, The Netherlands
C/EBPα	Rabbit	1/100	Santa Cruz Biotechnology
C/EBPβ	Mouse	1/100	Santa Cruz Biotechnology
Connexin 32	Mouse	1/100	Zymed
Cyp2E1	Rabbit	1/600	A gift from Professor Magnus Ingelmann-Sundberg, Karolinska Institute, Stockholm, Sweden
Glutamine synthetase (GS)	Mouse	1/300	Transduction Lab
Haptoglobin	Rabbit	1/200	Sigma
HNF4α	Rabbit	1/100	Santa Cruz Biotechnology
RXRα	Rabbit	1/150	Santa Cruz Biotechnology
Transferrin	Rabbit	1/100	DAKO, Ely, UK
UDP-glucuronosyltransferase (UGT)	Sheep	1/1000	Cypex Ltd., Dundee, UK
Vimentin	Mouse	1/100	Sigma

Secondary antibody			
Anti-mouse fluorescein Isothiocyanate (FITC) conjugated IgG	Horse	1/200	Vector Laboratories
Anti-rabbit FITC-conjugated IgG	Goat	1/200	Vector Laboratories
Anti-sheep FITC-conjugated IgG	Rabbit	1/200	Vector Laboratories
Anti-rabbit texas-red conjugated IgG	Rabbit	1/200	Vector Laboratories
Anti-rabbit tetramethylrhodamine isothiocyanate (TRITC) conjugated IgG	Swine	1/200	DAKO, Ely, UK

### 2.4 Western blotting

Protein extracts from cultured rat hepatocytes were prepared by lysing pre-washed cells with lysis buffer [20 mM HEPES (pH 7.6), 150 mM NaCl, 1 mM EDTA, 2 mM dithiothreitol (DTT) and 1% (v/v) Triton X-100] containing a 1/100 dilution of protease inhibitor cocktail (Sigma) for 10 min on ice. The cell lysates were then centrifuged at 13,000 rpm at 4 °C and supernatants were stored at −80 °C until further use. The protein concentration of the supernatant was determined using the Bio-Rad protein assay reagent according to the manufacturer's instructions. Total protein (10–15 μg) was mixed with same volume of 2× sample loading buffer [125 mM Tris–HCl (pH 6.8), 4% (v/v) SDS, 20% (v/v) glycerol, 0.2 mM DTT and 0.02% (v/v) bromophenol blue] and denatured by heating to 100 °C for 5 min. The proteins were then separated in 10% or 15% Criterion™ pre-cast Tris–HCl polyacrylamide gel (Bio-Rad) and transferred onto BioTraceNT^®^ nitrocellulose membrane (Pall Corporation, Pensacola, FL). The membrane was blocked with 5% (v/v) non-fat milk in 0.1% (v/v) PBS–Tween20 (Tween-20, was obtained from Sigma) at 4 °C overnight. The membrane was probed with primary and secondary antibodies (listed in Table 2) at room temperature for 2 and 1 h, respectively. Antibodies were diluted in 3% (v/v) non-fat milk in 0.1% (v/v) PBS–Tween and are listed Table 2. The signals were detected with the ECL™ Western blotting analysis system (Amersham) and developed on Hyperfilm™ (Amersham).

**Table 2:** 

	Species	Dilution factor	Supplier
Albumin	Rabbit	Primary 1/2000	Sigma
		Secondary 1/1000	Vector Laboratories

Haptoglobin	Rabbit	Primary 1/2000	Sigma
		Secondary 1/1000	Vector Laboratories

HNF1 α	Goat	Primary 1/2000	Santa Cruz Biotechnology
		Secondary 1/1000	Abcam, Cambridge, UK

HNF4 α	Goat	Primary 1/2000	Santa Cruz Biotechnology
		Secondary 1/1000	Abcam, Cambridge, UK

Transferrin	Rabbit	Primary 1/2000	DAKO, Ely, UK
		Secondary 1/1000	Vector Laboratories

α-Tubulin	Mouse	Primary 1/2000	Sigma
		Secondary 1/1000	Vector Laboratories

UDP-glucuronosyltransferase (UGT)	Sheep	Primary 1/2000	Cypex Ltd., Dundee, UK
		Secondary 1/1000	Abcam, Cambridge, UK

### 2.5 Qualitative and real-time RT-PCR

Total RNA was extracted from cultured rat hepatocytes using TRI reagent (Sigma, Poole, UK). The measurement of total extracted RNAs and reverse transcription were carried out as described previously (Li, Horb, Tosh, & Slack, 2005). Polymerase chain reactions containing the mixture of the same concentration of cDNA, 1.1X ReddyMix™ PCR Master Mix (ABgene, Surrey, UK) and 50 ng sense/antisense primers (listed in Table 3) were processed in a DNA thermal cycler using the following conditions: denatured at 94 °C for 1 min, amplification at 58 °C for 1 min and elongation at 72 °C for 1 min for indicated cycles. The samples were analysed in 1.2% agarose electrophoresis with 1 kb ladder marker (Invitrogen Life Technologies).

**Table 3:** 

	Forward primer	Reverse primer	Size of PCR product (bp)	GenBank accession No.	Cycles
CYP2B12	CGCATGGAGAAGGAGAAGTC	CCTCAGTGTTCTTGGGAAGC	352	X63545	28
CYP3A1	GGAAATTCGATGTGGAGTGC	AGGTTTGCCTTTCTCTTGCC	329	D29967	19
CYP7A1	CCTCCTGGCCTTCCTAAATC	GTCAAAGGTGGAGAGCGTGT	351	NM_012942	28
Tyrosine aminotransferase	GTCCATCGGCTACCTATCCA	CAGGACAGGATGGAACATT	492	NM_012668	24
Tryptophan 2,3-dioxygenase	GAGCAGGAGCAGACGCTATT	CACCTTGTACCTGTCGCTCA	498	NM_022403	19
Carbamoyl phosphate synthetase I	CGTCCAAGATTCCTTGGTGT	ATGGAAGAGAGGCTGGGATT	158	NM_017072	19
UDP-glucuronosyltransferase 1a (UGT1a)	ACACCGGAACTAGACCATCG	TGGAACCCCATTGCATATTC	152	NM_012683	19
Cyp reductase	GGCAAGGAGCTGTACCTGAG	ATGATGACAGGTGTGGTGGA	356	M110068	22
CAR	TGGCATGAGGAAAGACATGA	TCTGGTCCTCCATGGTAGG	350	NM_022941	24
PXR	GGTCTTCAAATCTGCCGTGT	GTTTCATGGCCCTTCTGAAA	103	AF151377	24
β-Actin	TCCGTAAAGACCTCTATGCC	AAAGCCATGCCAAATGTCTC	477	V01217	22

To obtain quantitative results, we performed real-time PCR using the Lightcycler system (Roche Diagnostics). The calibrators were the cDNA reverse transcribed from adult rat liver mRNA. The same amount of cDNA from calibrator and experimental samples were mixed with 1XSYBR^@^ Green Taq ReadyMix™ (Sigma), 50 ng sense and antisense primers and transferred into Lightcycler capillaries (Roche Diagnostics). The following conditions were used for the amplification of PCR products: denaturation at 95 °C for 30 s; amplification at 58 °C for 5 s followed by 72 °C for 20 s for 40–50 cycles; cool-down at 40 °C for 2 min. The fluorescence signal was detected at the same time point of each cycle. Data were presented as the normalised ratio, which is the target/reference ratio of the sample divided by the target/reference ratio of the calibrator by Lightcycler Relative Quantification software.

### 2.6 Periodic acid–Schiff's (PAS) staining

PAS staining was performed to detect glycogen. The cells were seeded onto coverslips and cultured for up to 4 weeks in KDS medium. The cells were then incubated with KDS plus 25 mM glucose for 24 h, fixed with 4% PFA and then permeabilised with 1% Triton X-100 at room temperature for 20–25 min. The cells were washed with tap water for 1–2 min and transferred to 1% periodic acid solution for 30 min. Next, the cells were washed with running tap water for 3 min and then incubated in the Schiff's reagent at room temperature for 30 min to develop. After washing in running tap water for 10 min, the slides were mounted in Gel/Mount mounting medium.

### 2.7 Urea cycle assays

Two colorimetric assays, designed to detect secretion of urea and activity of arginase, were performed on the cultured rat hepatocytes. The urea assay was based on a previously published protocol (Meng, Zhang, & Wu, 2004). Briefly, 100 μl of urea standards (0–50 μg/ml) and culture media collected over 24 h were incubated with 300 μl urease buffered solution (Sigma) at room temperature for 20 min. 600 μl of phenol nitroprusside, 600 μl of alkaline hypochlorite (both from Sigma) and 3 ml distilled water were then added, gently mixed and incubated at room temperature for 30 min. The absorbance of standards and samples were then measured at an optical density (OD) of 630 nm. Urea production was expressed as the amount accumulated in 24 h per culture dish (μg/dish/day).

The arginase assay was based on Corraliza, Campo, Soler, and Modolell (1994). Cultured rat hepatocytes were lysed in 0.1% (v/v) Triton X-100 containing a 1/100 dilution of protease inhibitor cocktail and shaken for 30 min. The lysate was then mixed with same volume of 25 mM Tris–HCl (pH 7)/5 mM MnCl_2_ and the enzyme was activated by incubation at 56 °C for 10 min. Activated lysate (25 μl) was then incubated with 25 μl of 0.5 M l-arginine at 37 °C for 1 h. The samples and the urea standards (0–500 μg/ml) were then incubated with 400 μl of an acid solution comprised of H_2_SO_4_:H_3_PO_4_:H_2_O at a ratio of 1:3:7 and 25 μl 9% (w/v, dissolved in 100% ethanol) *iso*-nitro-propiophenone (Sigma) at 100 °C for 45 min. The urea production was detected at an OD of 540 nm. For urea assay, the secreted urea was presented as the amount of urea per dish per day. The urea production from the hydrolysis of arginine by arginase was taken as a proportional representation of endogenous arginase activity in the arginase assay experiments. The results were normalised with total cellular protein and shown as the amount of urea produced per mg protein per day (μg urea production/mg total protein/day).

### 2.8 Image collection

Fluorescent images were collected using a Zeiss LSM 510 confocal microscope and figures compiled using Adobe Photoshop 7.0. For cell counting and PAS staining experiments, numbers of random fields were selected using the 40× objective lens of a Leica DMRB microscope. The cell numbers were visualised by 4,6-diamidino-2-phenylindole (DAPI) staining. Coverslips were incubated with DAPI (500 ng/ml) in PBS for 30 min at room temperature before being mounted on to slides in Gel/Mount mounting medium.

### 2.9 Statistical analysis

The data were expressed as mean ± standard deviation. Comparison of individual treatments was conducted using Student's *t*-test or one-way ANOVA analysis with Fisher's LSD pairwise comparison.

## 3 Results

### 3.1 Expression of hepatic differentiation markers in rat hepatocytes cultured in Williams’ medium E and KSFM

**Fig. 1:**
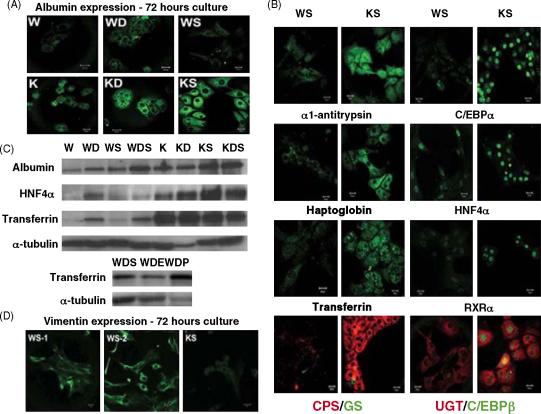
Expression of liver markers in short-term (72 h) culture of rat hepatocytes in Williams’ medium E and KSFM media. (A) Comparison of albumin expression in cultured rat hepatocytes after 72 h. Hepatocytes were cultured in Williams’ medium E or KSFM culture media with and without supplements. For details see Section 2. (B) Immunostaining of liver proteins in rat hepatocytes. Images in (A) and (B) were collected under the same conditions using a Zeiss LSM510 confocal microscope. (C) Western blotting analysis showing either the differential expression of albumin, transferrin or HNF4α in 72 h cultured rat hepatocytes or the expression of transferrin after 72 h of culture in WDS, WDE or WDP media. (D) Comparison of vimentin expression in WS or KS cultured rat hepatocytes after 72 h. Scale bars are 20 μm.

Despite numerous attempts, it has been very difficult to maintain hepatocytes in a well-differentiated state for more than a few days in culture without significantly changing the culture conditions. We therefore sought to develop a protocol for long-term maintenance of hepatic functions *in vitro* by the use of a simple medium. Several groups previously used KSFM for culture of oesophageal keratinocytes, corneal epithelial cells and foreskin keratinocytes (Andl et al., 2003; Chen, Chang, Lee, Javier, & Azar, 2002; Vallette et al., 1998). Moreover, Katsura et al. (2002) previously used KSFM medium along with serum to culture adult human hepatocytes (but in the absence of the EGF and PGE supplements). We tested the KSFM medium for maintaining the liver phenotype in primary rat hepatocytes in the absence and presence of EGF and pituitary gland extract. Adult rat hepatocytes isolated by the collagenase perfusion technique were allowed to attach in Williams’ medium E containing 10% serum and maintained for 6–10 h before changing to a serum-free defined Williams’ medium E or KSFM supplemented with Dex and/or EGF and pituitary gland extract. The initial measurements were taken for cultures at 72 h. Albumin expression was maintained in cells cultured in KSFM [either with Dex or EGF/pituitary gland extract (referred to as KD and KS, respectively, see Section 2)] in comparison to cells cultured in WD or WS (Fig. 1A). We then compared expression of other liver-specific markers including serum proteins (α1-antitrypsin, haptoglobin and transferrin), liver-enriched transcription factors (C/EBPα, C/EBPβ, HNF-4α and RXRα), enzymes associated with ammonia detoxification (GS and CPS) and Phase II metabolism (UDP-glucuronosyltransferase, UGT) in WS and KS. It was noted that KS maintained the hepatocyte heterogeneity in terms of populations of perivenous type GS and periportal type CPS-expressing cells (Fig. 1B). The results at 72 h showed that all the liver markers examined were maintained to a greater extent in KSFM-cultured conditions in comparison to Williams’ medium E (Fig. 1B and C).

To test the active component in the supplements, we added EGF and pituitary gland extract separately to the Williams’ medium E (Fig. 1C). Compared to EGF, pituitary gland extract had a greater effect on transferrin expression after 72 h of culture. However, we could not maintain the cells for more than 4–5 days, either with one or both the supplements. This result suggests that the KSFM medium itself must contribute to the maintenance of the hepatic phenotype, and it is not just an effect of supplements.

Vimentin is a cytoskeletal intermediate filament protein the expression of which has been associated with the de-differentiation of hepatocytes (Blaheta et al., 1998). We found that this was expressed in Williams’ medium E cultured isolated rat hepatocytes but was absent in KSFM-cultured cells after a 72-h culture period (Fig. 1D). This suggests that KSFM is preventing de-differentiation as well as maintaining differentiated functions.

**Fig. 2:**
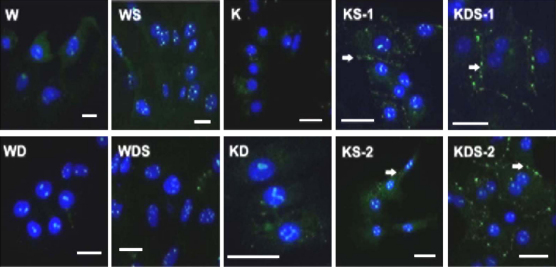
Connexin 32 expression in isolated rat hepatocytes. The expression of connexin 32 protein was detected in gap-junction-like arrays (arrows) in hepatocytes cultured with KS and KDS conditions but not Williams’ medium E after 72 h. Two examples of KS and KDS cultures are shown. Scale bars are 20 μm.

Connexin 32 (Cx32) is the predominant gap junction protein expressed in hepatocytes (Kumar & Gilula, 1986; Nicholson et al., 1987). Gap junction intracellular communication plays an important role in regulating cell survival and apoptosis (Krysko, Leybaert, Vandenabeele, & D’Herde, 2005), cell differentiation and proliferation (Cheng et al., 2004) and tumourigenesis (Luebeck, Buchmann, Schneider, Moolgavkar, & Schwarz, 2005). We examined the expression of Cx32 in rat hepatocytes cultured for 72 h in Williams’ medium E or KSFM media. As shown in Fig. 2, gap junction-like arrays of Cx32 were only detected in hepatocytes cultured in KS and KDS (Fig. 2).

### 3.2 Rat hepatocytes cultured in KDS medium retain their differentiated properties for up to 28 days

**Fig. 3:**
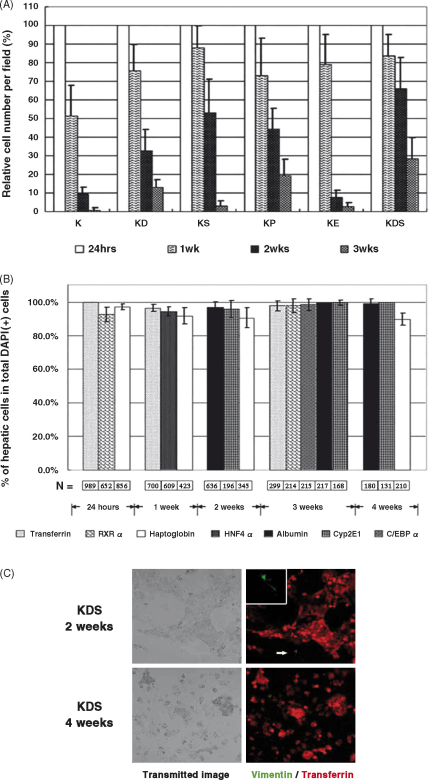
Survival of rat hepatocytes cultured in KSFM-medium. (A) The histogram describes the percentage of hepatocytes (based on DAPI-staining nuclei remaining from the 24-h culture point). Results are presented as means ± S.D. for at least five random fields in three different dishes. (B) Expression of hepatocyte proteins in DAPI-positive cells. Cells were cultured under KDS conditions and then fixed and immunostained for transferrin, RXRα, haptoglobin, HNF4α, albumin, Cyp2E1 or C/EBPα and counterstained with DAPI. The results are presented as a percentage calculated by dividing the number of cells expressing the appropriate hepatocyte protein by the total number of DAPI-positive cells. Results are shown as means ± S.D. for at least five random fields in three different dishes. Abbreviations are as indicated in Section 2. (C) The expression of transferrin (red) and vimentin (green) in hepatocytes maintained in KDS media for 2 or 4 weeks. The corresponding transmitted images are also shown.

Although the results for 3-day cultures with KSFM were encouraging, we wanted to know whether the medium could maintain the hepatic phenotype for longer culture periods. We began by examining the survival of hepatocytes under different culture conditions. Equal numbers of cells were seeded and cultured in six different KSFM culture conditions. The cell number at 24 h of culture was set as 100% and the percentages of hepatocytes at 1, 2 and 3 weeks were determined (Fig. 3A). In all cases there was a progressive loss of cells but the cells cultured in KDS exhibited the best survival rates, and we decided to use this combination for subsequent experiments. In addition, we also analysed the hepatic phenotype in the living cells by two different approaches. The first involved counting the number of DAPI-positive cells that also express liver proteins (Fig. 3B) or by immunostaining for the liver protein transferrin and collecting the corresponding transmitted light image to show the distribution of the cells (Fig. 3C). The results confirmed that more than 90% of the cells expressed liver proteins over 2 or 4 weeks of culture (Fig. 3B and C). Lastly, to determine whether KSFM prevents outgrowth of mesenchymal cells in long-term cultures, we co-stained for vimentin and transferrin after 2 and 4 weeks of KDS culture (Fig. 3C). At both time points we found only occasional vimentin positive cell (see insert in Fig. 3C) suggesting the KDS does indeed prevent outgrowth of mesenchymal cells.

**Fig. 4:**
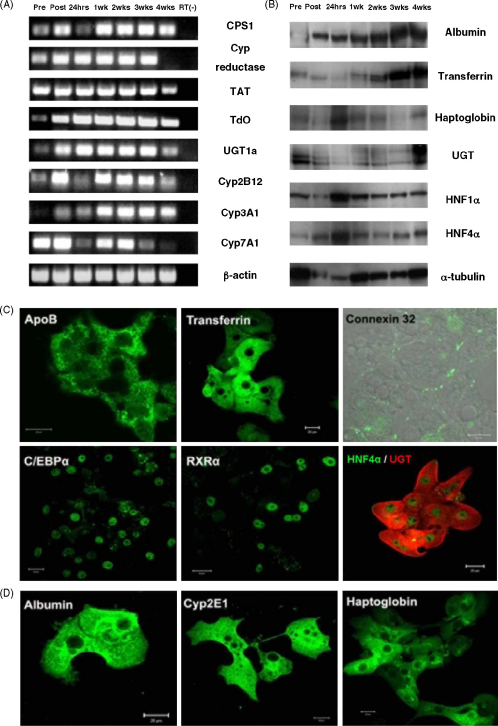
Long-term culture of rat hepatocytes in KDS medium. (A) RT-PCR investigation of the expression of carbamoylphosphate synthetase I (CPS1), cytochrome reductase, tyrosine aminotransferase (TAT), tryptophan di-2,3-oxygenase (TdO), UDP-glucuronosyltransferase 1a (UGT1a) and three cytochrome P450 genes (*Cyp2B12*, *Cyp3A1*, *Cyp7A1*); RT(−): no template control. (B) Western blotting detection of albumin, transferrin, haptoglobin, UGT, HNF1α and HNF4α in 24 h, 1, 2, 3 or 4 weeks cultured rat hepatoctyes. Pre and Post in (A) and (B) represent the mRNAs or proteins extracted from normal liver or freshly isolated hepatocytes, respectively. (C and D) Immunostaining analysis of liver proteins (Apolipoprotein B, transferrin, Cx32, C/EBPα, RXRα and HNF4α and UGT following 3 weeks (C) or 4 weeks (D) of culture under KDS conditions. Scale bar = 20 μm.

We determined the expression of liver markers in KDS-cultured rat hepatocytes using RT-PCR (Fig. 4A), Western blotting (Fig. 4B) and immunostaining (Fig. 4C and D) and compared the results to fresh liver and freshly isolated hepatocytes (designated ‘pre’ and ‘post’). For RT-PCR, the primers were designed towards messages of detoxification enzymes and enzymes involved in amino acid metabolism. All the transcripts were highly expressed in KDS-cultured rat hepatocytes up to 3 weeks and most were still present after 4 weeks of culture (Fig. 4A). Using Western blotting, hepatic proteins including haptoglobin and the liver-enriched transcription factor hepatocyte nuclear factor 1α (HNF1α) were maintained without diminution for at least 2 weeks (Fig. 4B). Furthermore, we also demonstrated that the expression of several liver proteins was maintained for at least 3 weeks. These include apolipoprotein B (ApoB), transferrin, albumin and Cyp2E1, C/EBPα, RXRα and HNF4α (Fig. 4C and D). In general, the expression of both transcription factors and differentiation products was similar to those in fresh liver.

### 3.3 Glycogen storage and urea cycle activity is preserved in KSFM-cultured rat hepatocytes

**Fig. 5:**
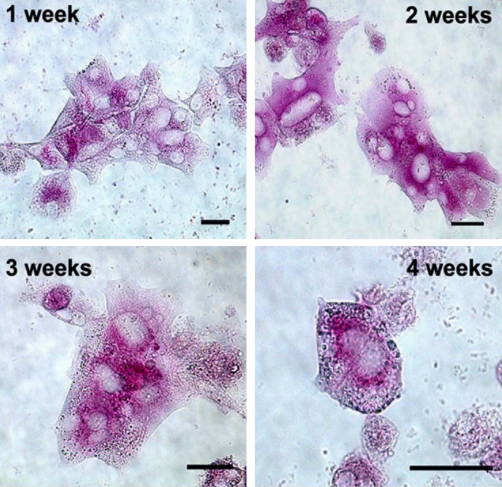
Glycogen storage in rat hepatocytes cultured in KDS medium. Glycogen was detected in rat hepatocytes by PAS staining at (A) 1 week; (B) 2 weeks; (C) 3 weeks; (D) 4 weeks of culture. Scale bar = 20 μm.

**Fig. 6:**
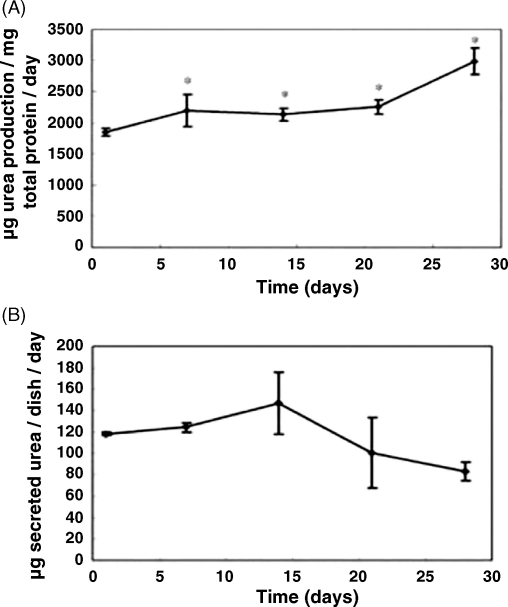
Time course of urea cycle activity in KDS-cultured rat hepatocytes. The arginase activity (A) and the concentration of secreted urea (B) in the KDS-cultured rat hepatocytes were determined as described in Section 2. Results are means ± S.D. For each hepatocyte isolation urea cycle analysis was performed on quadruplicate samples taken from four different culture dishes. At least three isolations were performed for each point. Statistical differences were determined between the 24 h result and subsequent time points by one-way ANOVA analysis with Fisher's LSD pairwise comparison. Significant differences (*P* < 0.05) are shown by ^*^.

To test whether hepatocytes cultured under different conditions maintained their functional capacity, we examined the potential to store glycogen and perform ureagenesis. We were able to detect glycogen in hepatocytes cultured with KDS for at least 4 weeks of culture (Fig. 5). We also investigated ureagenesis following culture of hepatocytes under KDS conditions. Assays were performed from 24 h up to 28 days of culture using either the detection of secreted urea (Fig. 6B), or by measurement of arginase activity (Fig. 6A). The results showed that both arginase activity and urea synthesis was generally maintained and are comparable to previously published values (Kang, Berthiaume, Nath, & Yarmush, 2004).

### 3.4 Hepatic metabolising enzymes are induced by phenobarbital treatment in KDS-cultured hepatocytes

**Fig. 7:**
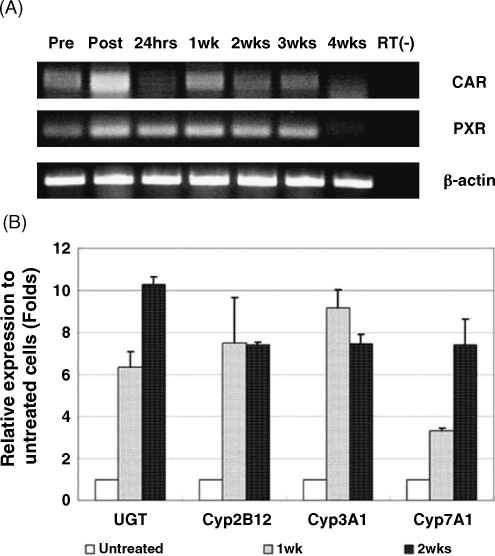
Phenobarbital-induced expression of liver metabolising enzymes. (A) Expression of CAR and PXR mRNA in KDS-treated rat hepatocytes. Cells were cultured for the times shown. Pre and Post represent the mRNAs extracted from normal liver or freshly isolated hepatocytes, respectively. (B) Hepatocytes were cultured with KDS for 7 or 14 days and then the medium was changed to KDS supplemented with and without 10 μM phenobarbital for another 3 days. The expression of UGT, Cyp2B12, Cyp3A1 and Cyp7A1 was performed by real-time RT-PCR. The results were displayed as the ratio of (Conc. [target gene (treated)]/Conc. [reference gene (treated)]):(Conc. [target gene (untreated)]/Conc. [reference gene (untreated)]). The calibrator is the mRNA from adult rat liver.

Phenobarbital is widely used as an inducer of Phase I and II metabolising enzymes in liver. Following treatment by phenobarbital, the transcription of phase I (cytochrome P450s) and phase II (UGT) detoxification enzymes are activated through the binding of CAR-RXR (CAR, constitutive active receptor; RXR, 9-*cis*-retinoic acid receptor) heterodimers with the *cis*-acting element (DR4 motifs, such as NR1 and NR2 domain on the enhancer of *Cyp2b10* gene) of target genes (Kakizaki et al., 2003). We initially examined the time course of expression of CAR and PXR (pregnane X receptor, an additional important nuclear receptor accounting for detoxification in hepatocytes) by RT-PCR in KDS-treated cells (Fig. 7A). The levels were well-maintained for 3 weeks and then started to drop off between 3 and 4 weeks of culture.

To examine the effect of phenobarbital on KDS-cultured rat hepatocytes, we analysed the mRNA expression of *UGT*, *Cyp2B12*, *Cyp3A1* and *Cyp7A1* by real-time RT-PCR. Rat hepatocytes were cultured for 7 or 14 days in KDS and then treated with and without phenobarbital for 3 days (total of 10 or 17 days culture). Fig. 7 shows that all the genes, including UGT and cytochrome P450s in the phenobarbital-treated cells, were upregulated 2–10 fold in comparison with control cells. The result demonstrates that hepatocytes cultured with KDS remain responsive to xenobiotics.

## 4 Discussion

Much effort has been devoted in the past in attempting to develop conditions suitable for the *in vitro* culture of rodent hepatocytes. Some culture media have been described for sustaining the differentiated state of hepatocytes but they only maintain function in the short term, i.e., 7 days or less (Michalopoulos, Bowen, Mule, & Luo, 2003; Miyazaki et al., 1998). Alternative approaches to the resolving the problem for human liver includes isolating and maintaining embryonic liver cells in culture or adult human hepatocytes (Lazaro et al., 2003, Runge et al., 2000). Our work here shows that KSFM medium with dexamethasone, EGF and pituitary gland extract is able to maintain the differentiated properties of rat hepatocytes for between 21 and 28 days. This statement is based on the sustained expression of liver-specific proteins (including liver-enriched transcription factors), the ability to induce drug metabolising enzymes, store glycogen and produce urea. The main benefit of KSFM medium is that it is relatively simple in comparison with some of the culture conditions reported by other groups. In previous studies, various supplements have been utilised to maintain hepatocytes in culture. These include growth factors (EGF and hepatocyte growth factor) (Michalopoulos et al., 2003, Miyazaki et al., 1998), differentiation-promoting chemicals (nicotinamide and DMSO) (Mitaka, 1998), metal ions (ferrous iron, copper, manganese or zinc) (Kojima et al., 1996), and hormones (e.g. glucocorticoid) (Shelly, Tynan, Schmid, Schutz, & Yeoh, 1989). In contrast, the present medium is relatively simple to prepare it is commercially available and the only supplements required are dexamethasone, EGF and pituitary gland extract. It is also worth noting that extracellular matrices (Rana, Mischoulon, Xie, Bucher, & Farmer, 1994) are not required. We have cultured hepatocytes in KDS media on collagen, fibronectin, matrigel and glass for 2 weeks and then immunostained for albumin. There were no differences showing that the KDS medium alone is sufficient to maintain albumin expression.

Liver-enriched transcription factors such as HNF1α, HNF4α and C/EBPα are important in the control of hepatic differentiation (Costa, Kalinichenko, Holterman, & Wang, 2003). Indeed over-expression of HNF4α by adenoviral infection prolongs the expression of liver-specific genes, cell viability and liver functions such as ammonia metabolism in cultured rat hepatocytes (Naiki, Nagaki, Asano, Kimata, & Moriwaki, 2005). In addition, C/EBPβ has been shown to be a master regulator for the formation of transdifferentiated hepatocytes from pancreatic-derived cells (Kurash, Shen, & Tosh, 2004; Shen et al., 2000, Tosh et al., 2002). When we examined the expression of the liver-enriched transcription factors we found that C/EBPα and β, HNF4α and RXRα expression were reduced within 3 days of culture with Williams’ medium E but were maintained when the cells were cultured in KDS. This observation suggests that loss of liver-enriched transcription factors is probably the cause of the loss of the differentiated hepatic phenotype.

Recently, it was suggested that loss of connexin expression might be used as a prognostic marker for hepatocellular carcinoma (Sheen et al., 2004). The presence of connexin proteins in liver is associated with normal liver function. The induction of Cx32 and Cx26 proteins in DMSO-containing primary mouse hepatocytes cultures was also observed and confirmed the importance of connexin proteins in differentiating hepatocytes (Stoehr & Isom, 2003). In the present study, Cx32 was shown to be expressed in hepatocytes maintained in KDS culture medium for up to 3 weeks (Fig. 4C). The persistent expression of Cx32 may provide further evidence of the efficacy of KDS as a medium for primary hepatocyte culture.

Although the KDS mixture does not constitute a defined medium, because of uncertainty about the precise composition of the pituitary extract, our results do show that KSFM, the EGF and the PGE all have a part to play in the maintenance of differentiated hepatocytes in culture. We note that PGE alone has previously been shown to maintain the differentiation status of mammary carcinoma cells (Kano-Sueoka et al., 1979, Platica et al., 1992).

In summary, we describe a simple, novel system for maintaining rat hepatocytes in culture and show that the differentiated phenotype can be maintained for long periods in culture. This method may provide a standard system for studies on drug metabolism and toxicity testing without the problem of overgrowth of mesenchymal cells. Primary hepatocytes are a better model for the liver *in vitro* than hepatoma cell lines, but they also provide a cheaper alternative and a more controllable system than the intact animal. The method may also have potential for reducing the number of animals used for this type of work. Finally, we believe that this long-term differentiated culture method could also pave the way for bioengineering applications such as the development of a bioartificial liver.
